# Evaluation of Prescription Practices Using WHO/INRUD Indicators and Determinants of Polypharmacy at a Kenyan County Referral Hospital

**DOI:** 10.1155/adpp/8031546

**Published:** 2025-10-31

**Authors:** Jeremiah Kayioni, Eric Guantai, Margaret Oluka, Faith Okalebo

**Affiliations:** ^1^ Department of Pharmacy, Kapenguria District Hospital, Kapenguria, Kenya; ^2^ Department of Pharmacology, Clinical Pharmacy and Pharmacy Practice, University of Nairobi, Nairobi, Kenya, uonbi.ac.ke

**Keywords:** prescribing practices, rational drug use, WHO indicators

## Abstract

**Introduction:**

Improving treatment standards by auditing care quality using WHO and INRUD drug‐use indicators is essential in low‐ and middle‐income countries. This study aims to assess prescription practices at KCRH General Outpatient Clinics and identify risk factors for polypharmacy.

**Methods:**

This retrospective descriptive cross‐sectional study analyzed prescriptions dispensed in 2022 at the KCRH outpatient pharmacy. Using a stratified random sampling, prescriptions were reviewed using a WHO/INRUD‐based data abstraction form. Core drug‐use indicators were determined, and logistic regression analyses were performed using STATA Version 14.

**Results:**

Of the 920 prescriptions, 69.2% were for adults and 57.0% for females. Analgesics (32.3%) and antibiotics (29.0%) were most frequently prescribed. Overall, 84.1% of the prescriptions contained antibiotics, 8.7% injectables, 97.6% generics, and 95.6% were from the KEML. The mean number of drugs per prescription was 2.7. Antibiotic prescribing was associated with lower odds of polypharmacy (aOR: 0.18; 95% CI: 0.10–0.42).

**Conclusion:**

Interventions to promote rational antibiotic use are necessary, including educating healthcare providers and patients about the risks of antibiotic overuse.

## 1. Introduction

Rational drug use involves the use of medications appropriate to patients’ clinical needs, in correct doses, for adequate periods, and at the lowest cost [1]. The World Health Organization (WHO) estimates that inappropriate prescribing practices affect healthcare systems globally, particularly in low‐ and middle‐income countries (LMICs) [2]. These practices have been attributed mainly to increased morbidity, mortality, and healthcare costs [3]. In Sub‐Saharan Africa, where healthcare resources are limited and medication costs represent a substantial portion of household expenditure, irrational drug‐use patterns can have devastating consequences for both individual patients and health system sustainability [4]. Common forms of irrational prescribing include polypharmacy, inappropriate antibiotic use, and overuse of injectable medications, as well as prescribing outside national essential drug lists [5]. These practices contribute to antimicrobial resistance, increased treatment costs, and adverse drug reactions that could otherwise be prevented through rational prescribing [5]. The economic burden of irrational prescription is substantial, and healthcare expenditure on medications represents approximately 25% of global health spending [6]. However, due to cost and availability barriers, many essential medicines remain inaccessible to nearly half the world’s population [7].

In LMICs, including Kenya, the challenge of irrational prescribing is compounded by limited resources, high disease burden, and variable adherence to standard treatment guidelines (STGs). Studies from different parts of the country have reported high rates of antibiotic prescribing, suboptimal use of generics, and frequent polypharmacy [8–10]. Such practices not only undermine rational drug use but also increase financial strain on patients and the health system.

The WHO and the International Network for Rational Use of Drugs (INRUD) have developed standardized core indicators to evaluate prescribing patterns in healthcare settings [11]. These validated indicators include the average number of drugs per prescription, the percentage of generic drugs prescribed, the rate of prescriptions containing antibiotics, the percentage containing injectable drugs, and the percentage of prescribed drugs from national essential drug lists [12]. These indicators provide a systematic framework for identifying problematic prescribing patterns and monitoring improvements in rational drug use.

Investigation of prescription patterns can improve prescription quality and rationality of drug use [12]. However, evidence on prescribing practices in the country referral hospital remains limited at the institutional level. In particular, no published study has evaluated prescribing patterns at Kapenguria County Referral Hospital (KCRH). Bridging this evidence gap is essential to identify context‐specific challenges, evaluate against WHO/INRUD standards, and guide interventions aimed at improving rational drug use. Therefore, this study aims to evaluate the quality of prescribing in KCRH using the WHO/INRUD methodology. The study’s findings will be pivotal in informing policy formulation on drug‐use and prescription patterns in the county and, by extension, the country.

## 2. Materials and Methods

### 2.1. Study Design

The study utilized a retrospective descriptive cross‐sectional design and analyzed historical prescription data from the KCRH outpatient clinic for 2022.

### 2.2. Study Area

The study was conducted at the KCRH general outpatient clinic. The outpatient clinic is divided into a general adult outpatient clinic and a general pediatric outpatient clinic. KCRH is located in West Pokot County in the Rift Valley region. West Pokot County borders Baringo County to the East, Turkana County to the North, Trans‐Nzoia and Elgeyo Marakwet to the South, and Uganda to the West. [13]. It is a government Level 4 county hospital that offers inpatient and outpatient health services with a bed capacity of 286 [14].

### 2.3. Study Population and Inclusion Criteria

The study included prescriptions from KCRH’s general outpatient clinics prescribed in 2022, which were received and stored at the hospital’s records department. Prescriptions were included if dispensed at the outpatient pharmacy of KCRH from general adult and pediatric outpatient clinics, while prescriptions from specialized clinics and those intended to manage chronic diseases were excluded. The study also included facility guides for prescribing, such as the availability of the Kenya Essential Medicines List (KEML) (2019), clinical guidelines, and key medicines.

### 2.4. Determination of Sample Size

Since the study was cross‐sectional, the population was less than 10,000. Fisher’s formula was used to determine the minimum sample size [15], where 5% was used as the margin of error, and the expected prevalence of rational drug use in primary public health facilities in Kisii County (0.76) was used [9]. The calculated minimum sample size was 280 prescriptions. However, as per the terms of the WHO, a cross‐sectional study designed to describe the prescribing patterns in a given hospital facility must have a sample size of at least 600 [16].

### 2.5. Sampling Procedure

Only patient prescriptions meeting the inclusion criteria for the year 2022 were considered for sampling. Since handwritten prescriptions were unavailable, only electronic prescriptions were used. Data from the electronic records were accessed from the Check Health Information System 2020 database on July 27, 2023, and the sampling frame comprised 63,410 prescriptions. Prescriptions were randomly sampled. To increase the statistical power, 1200 prescriptions were sampled, arranged in chronological order, and a table of random numbers was employed to select prescriptions.

### 2.6. Data Collection

Data collection utilized a standardized data abstraction form adapted and refined from the WHO/INRUD tool [16]. Content validity was ensured through expert judgment, where the supervisors assessed its capacity to yield accurate data. Two trained pharmaceutical technologists served as research assistants after undergoing a comprehensive one‐day training session on data extraction from the Check Health Information System 2020 database. Patient‐specific demographic variables (age and sex), prescriber indicators (cadre and sex), prescribed medication, prescription indicators, facility data, and prescription completeness were all captured.

### 2.7. Study Variables and Definition

The study’s outcome variable focused on drug prescribing practices by healthcare providers, assessed through five indicators. The first indicator measured the number of drugs per encounter. The second indicator evaluated the percentage of drugs prescribed using their generic names. The third indicator gauged the percentage of antibiotics prescribed. The fourth assessed the percentage of injections prescribed, while the last indicator assessed the percentage of drugs prescribed from the KEML [17]. Availability of the KEML 2019 list and formulary or clinical guidelines for practitioners was assessed by checking their presence and accessibility within KCRH. Polypharmacy is defined as the regular use of 5 or more medications at the same time. The independent variables in the study included prescriber and patient characteristics.

### 2.8. Data Management and Quality Assurance

The personal computers and laptops used to store the raw data were password‐protected, and all data entered into Microsoft Excel were coded and encrypted. The principal investigator safely stored all the flash disks and hard copies.

### 2.9. Data Analysis

Continuous variables were summarized using means and standard deviation, while frequencies and percentages were used to summarize categorical variables. Inferential data analysis was performed using chi‐square tests and one‐sample *t*‐tests, and bivariate and multivariate logistic and linear regression analyses were carried out to identify factors associated with antibiotic prescribing and polypharmacy, respectively. Data were analyzed using STATA Version 14 software. The level of significance was set at 0.05.

### 2.10. Ethical Considerations

The study received ethical approval from the Kenyatta National Hospital/University of Nairobi Ethics and Research Committee (KNH/UoN‐ERC) under approval number P106/022023. No primary data were collected directly from the patients in this study, so the need for consent was waived.

## 3. Results

**Figure 1:**
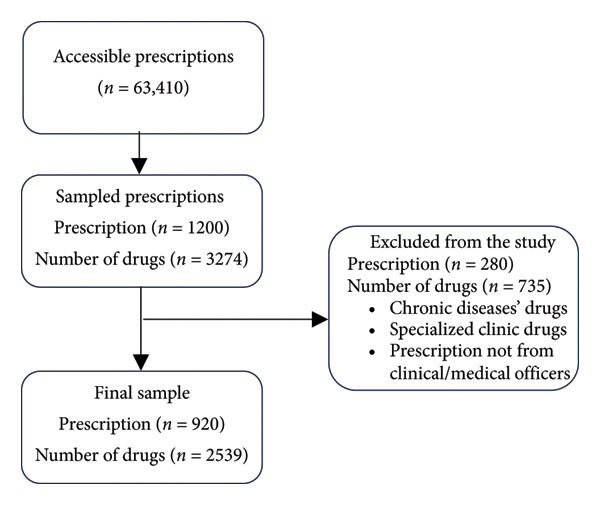
Sample size flowchart.

A total of 1200 prescriptions were screened for eligibility in the study. Out of the 1200 prescriptions, 280 (23.3%) from specialized clinics and those intended to manage chronic diseases were dropped as per the exclusion criteria. Therefore, 920 (76.7%) were analyzed, corresponding to a total of 2539 drug encounters. The reasons for exclusion are summarized in Figure 1.

### 3.1. Patient and Prescriber Characteristics

More than half of the patients were adults, 637 (69.2%), and 524 (57.0%) were females. Among the prescribers, 446 (48.5%) were males, and 858 (93.3%) were clinical officers, as shown in Table 1.

**Table 1:** Demographic characteristics of patients and prescribers at the outpatient clinics of Kapenguria County Referral Hospital (*n* = 920).

Characteristics	Variable	Frequency (*n* = 920)	Percentage (%)
*Patients*			
Age (years)	Pediatrics (≤ 12 years)	283	30.8
	Adults (> 12 years)	637	69.2
Sex	Females	524	57.0
	Males	396	43.0

*Prescribers*			
Sex	Males	446	48.5
	Females	474	51.5
Cadre	Clinical officers	858	93.3
	Medical doctors	62	6.7

**Figure 2:**
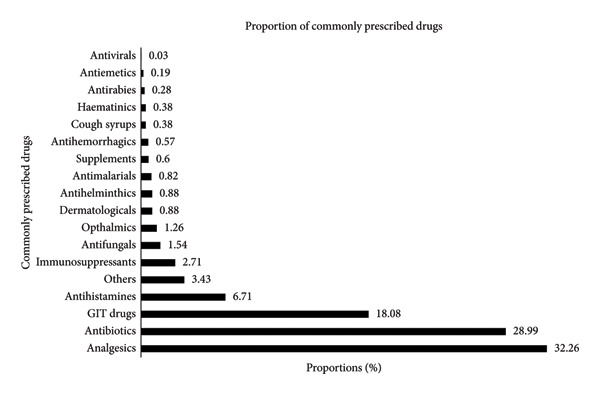
The most commonly prescribed drugs at the outpatient clinics of Kapenguria Country Referral Hospital.

Of the 2539 drugs prescribed, the most frequently prescribed drugs were analgesics (1,024, 32.3%), antibiotics (920%, 29.0%), and GIT drugs (574, 18.1%). Conversely, antiemetics and antivirals were the least prescribed, each accounting for only 6 (0.2%) and 1 (0.0%) of the prescribed drugs, respectively, as shown in Figure 2.

Access antibiotics such as amoxicillin and amoxicillin–clavulanate dominate the prescribing practice in Kapenguria. However, watch antibiotics such as cefixime and azithromycin are still frequently used (Table 2).

**Table 2:** Categorization of commonly prescribed antibiotics as per the WHO AWaRe classification (2023).

No	Commonly prescribed	Percentage (%)	WHO AWaRe classification
1	Amoxicillin	25.9	Access
2	Amoxicillin–clavulanate	22.4	Access
3	Azithromycin	5.0	Watch
4	Cotrimoxazole	9.4	Access
5	Flucloxacillin	9.1	Access
6	Cefixime	7.6	Watch
7	Ciprofloxacin	5.8	Watch
8	Clarithromycin	4.1	Watch
9	Doxycycline	1.2	Access
10	Erythromycin	1.0	Watch
11	Levofloxacin	3.9	Watch
12	Ampicillin	0.7	Access
13	Nitrofurantoin	2.1	Access
14	Cefuroxime	0.6	Watch
15	Clindamycin	0.2	Access
16	Ceftriaxone	1.1	Watch

### 3.2. Assessment of Prescribing Patterns Using the WHO Prescribing Indicators

From Table 3, the mean number of drugs per prescription was 2.7 (SD 1.0). The percentage of prescriptions with antibiotics was 84.1%, and the percentage with injectables was 8.7%. The percentage of drugs prescribed by a generic name was 97.6%. Out of the 2539 drugs assessed across encounters, 2427 (95.6%) drugs were prescribed from the KEML.

**Table 3:** Prescription indicators using WHO core indicators at the outpatient clinics of Kapenguria County Referral Hospital.

Prescription indicator assessed	Total drugs/encounter	Mean (SD)/percent (%)	WHO optimal value
Mean number of drugs per encounter	2539	2.7 ± 1.0	1.6–1.8
Percentage of encounters with antibiotics	774	84.1%	20.0%–26.8%
Percentage of encounters with injection	80	8.7%	13.4%–24.1%
Percentage of drugs prescribed by generic name	2477	97.6%	100%
Percentage of drugs prescribed from ^∗^KEML (2019)	2427	95.6%	100%

^∗^Kenya Essential Medicine List.

### 3.3. Availability of Key Drugs and Documents to Facilitate Prescribing

Table 4 presents an inventory of essential drugs and their strengths. All essential medications except for griseofulvin tablets were available. The availability of essential drugs, including those listed in the KEML, was 93.8% as compared to the optimal value of 100%.

**Table 4:** Availability of key medicines and documents at Kapenguria Country Referral Hospital.

Essential drug	Strength	Availability of key essential drugs
Amoxicillin capsules	250, 500 mg	✓
Cetirizine tablets	5, 10 mg	✓
Paracetamol tablets	125, 500 mg	✓
Ibuprofen tablets	200, 400 mg	✓
Cotrimoxazole tablets	480 mg	✓
ORS + zinc sulfate		✓
Albendazole tablets	400 mg	✓
Metronidazole tablets	200, 400 mg	✓
Benzyl penicillin injection	1, 5 MU	✓
Clotrimazole pessaries	100, 500 mg	✓
Artemether lumefantrine (AL)	20 + 120 mg	✓
Artesunate injection	60 mg	✓
Nystatin oral drops	100,000 IU	✓
Griseofulvin tablets	125, 250 mg	×
Fluconazole capsules	200 mg	✓
Ferrous sulfate tablets	200 mg	✓

Percentage availability of key drugs		93.8%
Percentage availability of KEML copies		100%

*Note:* “×,” not available; “✓,” available.

### 3.4. Determinants of Polypharmacy

Factors such as antibiotic prescription, injection encounter, prescriber sex, and cadre were identified to be key determinants of polypharmacy from the multivariate logistic regression. Male prescribers and the clinical officer had a two‐fold increase in the odds of polypharmacy (aOR: 2.143, 95% CI: 1.013 and 4.533, *p* = 0.046; aOR: 2.187, 95% CI: 1.630 and 2.935, *p* < 0.001), respectively, as compared to the female prescribers and medical officers. Furthermore, patients with antibiotic prescriptions had an 88% reduction in odds of polypharmacy (aOR: 0.182, 95% CI: 0.104 and 0.421, *p* = 0.006) as compared to patients without antibiotic prescriptions (Table 5).

**Table 5:** Bivariate and multivariate logistic regression for polypharmacy determinants at the Kapenguria County Referral Hospital outpatient clinics.

Characteristics	Variable	^a^cOR (95% CI)	*p* value	^b^aOR (95% CI)	*p* value
Patient age	Pediatric	Ref		Ref	
	Adults	0.778 (0.388, 1.558)	0.479	0.583 (0.272, 1.252)	0.167

Patient sex	Female	Ref		Ref	
	Male	0.836 (0.422, 1.165)	0.608	0.769 (0.365, 1.619)	0.490

Antibiotic prescription	No	Ref		Ref	
	Yes	6.867 (1.933, 8.626)	**0.051**	0.182 (0.104, 0.421)	**0.006**

Injection encounter	No	Ref		Ref	
	Yes	8.531 (4.142, 17.571)	**< 0.001**	16.50 (7.405, 36.8)	**< 0.001**

Prescribers’ sex	Females	Ref		Ref	
	Males	2.189 (1.081, 4.433)	**0.029**	2.143 (1.013, 4.533)	**0.046**

Prescribers’ cadres	Clinical officers	Ref		Ref	
	Medical officers	0.699 (0.592, 0.825)	**< 0.001**	2.187 (1.630, 2.935)	**< 0.001**

*Note:* Bold values represent statistically significant variables.

^a^cOR, crude odds ratio.

^b^aOR, adjusted odds ratio.

## 4. Discussion

Based on the study findings, the mean number of drugs prescribed per patient encounter was 2.7 and 2.6 for both adults, females and males, respectively. However, the averages were higher among pediatric patients at 2.9. These rates were above the WHO/INRUD optimal range of 1.6–1.8 [16], indicating overprescription. This average is almost similar to what a study in a public hospital in Kenya found (2.83) [10]. Other studies performed in Kenya [9], Uganda [18], Egypt [19], and Ghana [20] found a higher than the recommended optimal mean of 2.9, 2.6, 2.5, and 4.8, respectively. These high rates of drugs prescribed per patient encounter could be attributed to the unavailability of STGs, inadequate continuous medical education programs, and incompetent prescribers [21]. On the other hand, studies performed in different parts of Ethiopia [22–24] and Eritrea [25] reported average numbers within the WHO/INRUD range. The variance in the reported numbers across different studies could be attributed to variations in study sites, settings, and designs. Moreover, the differences in prescribing patterns among various medical disciplines could also be attributed to these variations [25].

From the study findings, the percentage of drugs prescribed by their generic names was estimated at 97.6%. This is slightly below the optimal percentage recommended by the WHO/INRUD, which is 100% [16]. This value was, however, higher in comparison to that of public hospitals in Kenya, with Thika at 64% [10], Makueni County at 45.5% [8], Kisii County at 27.7% [9], and Mbagathi Hospital at 25.6% [26]. However, this study reported lower values compared to what was reported in South Ethiopia (99%) [23] and Mozambique (99%) [27]. The lower figure observed in this study, compared to the WHO’s recommended standards, may be linked to several factors. These could include prevailing preference for branded medications over their generic counterparts, the extensive promotional efforts conducted by pharmaceutical companies, or the absence of a national policy promoting the prescription of generic drugs.

In KCRH, the percentage of encounters with an injection prescribed was 8.7%, lower than the WHO optimal value (13%–24%) [16]. Our value was slightly higher compared to similar studies performed in Uganda (5.4%) [18], Eritrea (6.6%) [25], and India (5.7%) [28]. Conversely, our value was much lower in comparison to studies performed in other LMICs, such as Bangladesh (38.1%) [29], Kisii County (Kenya) (24.9%) [9], Sri Lanka (30.1%) [30], Northwest Ethiopia (9.5%) [31], and Libya (30.4%) [32]. One reason for the lower use of injections could be prescribers’ strong preference for the oral route, since injectable formulations carry higher risks of disease transmission, increased patient noncompliance, and greater costs and generally require trained personnel for proper administration. Most (95.6%) of the drug prescriptions in KCRH were performed from the KEML (2019). This was slightly below what is recommended by the WHO/INRUD (100%). Other studies reported comparable findings: 100% in Ethiopia [31] and 96.7% in Tanzania [33]. However, this percentage was higher compared to studies done in Mbagathi Hospital (Kenya) (72.2%) [26], Makueni County Referral Hospital (Kenya) (89.1%) [8], Kampala (Uganda) 78.9% [18], and Libya (82.8%) [32]. KCRH had a copy of the KEML (2019); thus, most prescriptions were made from the list.

The study found increased odds of polypharmacy among male prescribers and medical officers. While male and female physicians are known to differ in patient interaction and communication styles, little research has explored how prescriber gender influences prescribing patterns [34]. Two recent studies suggested that female prescribers tended to prescribe fewer drugs, especially to female patients, though these findings were not statistically significant [35, 36], contrasting with the current study’s results.

### 4.1. Strengths and Limitations

The strengths of the present survey are that it provides a comparative analysis with findings from other regions and countries, highlighting similarities and differences in prescription practices. Moreover, it includes a comprehensive review of over 600 prescriptions from KCRH pharmacies. However, this study is limited in its generalizability as it was conducted in a single institution. Its cross‐sectional design restricts causal inferences, offering only a snapshot of antibiotic prescribing practices. The lack of indication data for prescribed antibiotics and the exclusion of patient care indicators further hinder the assessment of medication appropriateness and overall quality of care. In addition, the study documents the count of coprescribed medications with antibiotics but does not analyze individual coprescribed drugs.

## 5. Conclusion

Most of the prescribing indicators exhibited significant deviations from the recommended optimal values by WHO/INRUD, suggesting the presence of irrational drug usage practices, including the inappropriate use of antibiotics. Factors such as prescriber qualification, patient sex, the number of drugs, and prescriptions involving generic and injections were identified as associated with antibiotic prescribing. The findings of this study underscore the necessity for interventions to promote the rational use of antibiotics. Various strategies can be recommended to promote rational drug utilization, including establishing drug and therapeutic committees and STGs; offering targeted ongoing education; ensuring the availability, accessibility, and affordability of high‐quality drugs; conducting drug‐use evaluations; and disseminating drug bulletins.

**Nomenclature**
DDD: Defined daily dose
DTC: Drugs and Therapeutic Committee
EDL: Essential drug list
KEML: Kenya Essential Medicines List
INRUD: International Network for Rational Use of Drugs
KCRH: Kapenguria County Referral Hospital
LMICs: Low‐ and middle‐income countries
OPD: Outpatient department
PI: Principal investigator
WHO: World Health Organization

## Ethics Statement

Permission to conduct the study was provided by KNH UoN‐ERC via approval number: P106/02/2023.

## Conflicts of Interest

The authors declare no conflicts of interest.

## Funding

This study did not receive any form of funding or financial assistance.

## Data Availability

The data that support the findings of this study are available on request from the corresponding author. The data are not publicly available due to privacy or ethical restrictions.
